# Non-neutralizing Antibodies Targeting the V1V2 Domain of HIV Exhibit Strong Antibody-Dependent Cell-mediated Cytotoxic Activity

**DOI:** 10.1038/s41598-017-12883-6

**Published:** 2017-10-04

**Authors:** Luzia M. Mayr, Thomas Decoville, Sylvie Schmidt, Géraldine Laumond, Jéromine Klingler, Camille Ducloy, Seiamak Bahram, Susan Zolla-Pazner, Christiane Moog

**Affiliations:** 1 INSERM U1109, Fédération de Médecine Translationnelle de Strasbourg (FMTS), Université de Strasbourg, Strasbourg, France; 2 Division of Infectious Diseases, Icahn School of Medicine at Mount Sinai, New York, New York, USA; 3 Vaccine Research Institute (VRI), Créteil, France

## Abstract

The development of an effective vaccine against HIV-1 has proven to be challenging. Broadly neutralizing antibodies (bNAbs), whilst exhibiting neutralization breadth and potency, are elicited only in a small subset of infected individuals and have yet to be induced by vaccination. Case-control studies of RV144 identified an inverse correlation of HIV-1 infection risk with antibodies (Abs) to the V1V2 region of gp120 with high antibody-dependent cellular cytotoxicity (ADCC) activity. The neutralizing activity of Abs was not found to contribute to this protective outcome. Using primary effector and target cells and primary virus isolates, we studied the ADCC profile of different monoclonal Abs targeting the V1V2 loop of gp120 that had low or no neutralizing activity. We compared their ADCC activity to some bNAbs targeting different regions of gp120. We found that mAbs targeting the V1V2 domain induce up to 60% NK cell mediated lysis of HIV-1 infected PBMCs in a physiologically relevant ADCC model, highlighting the interest in inducing such Abs in future HIV vaccine trials. Our data also suggest that in addition to neutralization, lysis of infected cells by Abs can effectively participate in HIV protection, as suggested by the RV144 immune correlate analysis.

## Introduction

A strong antibody (Ab) response directed against gp120 and gp41 envelope proteins is mounted in essentially all HIV-1 infected individuals. However, these Abs have limited neutralization capacity against the constantly mutating virus. Indeed, only a small subset of infected subjects produce broadly neutralizing antibodies (bNAbs) that possess neutralization breadth and potency against different HIV-1 subtypes^1,2^. The monoclonal bNAbs generated from these patients however display special features such as high rates of somatic mutation and long complementary-determining region 3 (CDR3) sequences, making them difficult to be induced by vaccination.

Follow-up analysis of the immune correlates of RV144, the Thai HIV-1 vaccine trial - which reported modest protection (estimated 31.2% vaccine efficacy) - showed a correlation in vaccinees between reduced infection rates and high levels of Abs targeting the V1/V2 region of the HIV gp120 envelope glycoprotein. Further analyses indicated that, in the presence of low IgA Env Abs, Ab-dependent cellular cytotoxicity (ADCC) inversely correlated with a reduced rate of infection^3,4^. Neutralizing activity did not contribute to the protective effect of RV144^5^.

The V1V2 domain is immunogenic and Abs targeting this region are induced in most HIV-1 infected patients^6^. It is located at the distal apex of the trimeric HIV-1 Env spike, where it is accessible to Abs^7,8^. The region assumes a five-stranded beta barrel structure and contains the LDV/I motif that has been shown to bind to the α4β7 integrin, important for T cell homing^9,10^. Interestingly, α4β7 mAb treatment of macaques that lead to sustained SIV virological control also promoted V2 antibody responses, recognizing the same region identified in a sieve analysis for immune correlates of reduced risk in the RV144 vaccine trial^11^.

A set of mAbs, termed V2i mAbs, was isolated from HIV-1 clade B-infected patients and recognize a conformation-dependent epitope surrounding the α4β7 integrin binding site of the V1V2 domain^12–15^. These V2i mAbs display numerous physiochemical similarities to the Abs elicited in RV144 such as weak neutralizing activity and overlapping epitope regions. However, their ADCC activity was not yet assessed. Abs targeting this region are commonly induced by HIV-1 infection and are also elicited by vaccination^3,6,16,17^. This makes them a promising tool for HIV-1 vaccine research.

ADCC activity is characterized by the interaction of the Fc region of an immunoglobulin, bound via its Fab part to Env on infected cells, and to Fc receptors expressed on the surface of effector cells, such as NK cells. This triggers the release of cytotoxic granules containing perforin and granzymes, leading to the death of the antibody-bound infected target cell. In this regard, ADCC is a complex multistep process. Several user-optimized assays have been developed with different target cells, effector cells and end-point read-outs to detect ADCC *in vitro*, but it is not known which assay shows the best correlation with *in vivo* protection.

Noteworthy, assays using HIV-1 infected cells showed that CD4 binding modifies the expression of ADCC epitopes^18^ and lysis of primary HIV-infected T cells was shown to be enhanced by the addition of CD4 mimetics^19^. These results imply that some HIV epitopes may be masked on infected cells, therefore allowing the virus to escape from ADCC in physiologically relevant *in vivo* situations.

Although difficult to compare to each other, these assays have shown that ADCC may effectively participate in protection against HIV. In addition to the RV144 immune correlate analysis, high levels of ADCC Abs have been associated with slow disease progression in both HIV and simian immunodeficiency virus (SIV) models^20–22^. Local application of Abs directed against the principal immunodominant domain (PID) of gp41 and exhibiting ADCC and Fc-mediated inhibitory activities *in vitro* were shown to decrease the viral load set point and the number of transmitted/founder viruses in macaques challenged experimentally^23,24^. ADCC activity was also significantly higher in HIV elite controllers, a group of rare HIV-1 infected patients who were able to maintain viremia below detectable limits^25^. In the context of mother-infant transmission, high ADCC activity in maternal breast milk reduced HIV infection of the infant via breastfeeding and decreased the mortality risk of the infected infants^26,27^. These and additional studies have pointed towards ADCC as an important mechanism to target HIV-1 *in vivo* ^28–30^.

Recently, Bruel *et al*. reported a lack of ADCC activity in non-neutralizing anti-HIV-1 Abs, using re-activated T cells from HIV-1 infected patients. The study included non-neutralizing Abs targeting the V3 crown, CD4 binding site, and the PID site of gp41. Non-neutralizing Abs targeting V2 epitopes were not tested^31^.

The present study was performed to assess and compare the ADCC capacity of mAbs using an assay that directly measures the lysis of infected primary CD4 T cells and is therefore physiologically more relevant than assays using cell lines. Abs to different regions of gp120 were included, but the focus was on anti-V1V2 mAbs, isolated from HIV-1 infected patients and similar to Abs induced by RV144, with no or low neutralizing activity. In addition, other anti-V1/V2 Abs and well-characterized bNAbs were used. Our data suggest that mAbs targeting the V1V2 domain of gp120 show exceptional ADCC activity using this assay and are highly relevant Abs for inducing ADCC activity.

## Results

### Non-neutralizing V2i mAbs induce stronger NK cell-mediated lysis of HIV-1_SF162_ infected CD4 T cells than a panel of three broadly neutralizing mAbs

**Figure 1:**
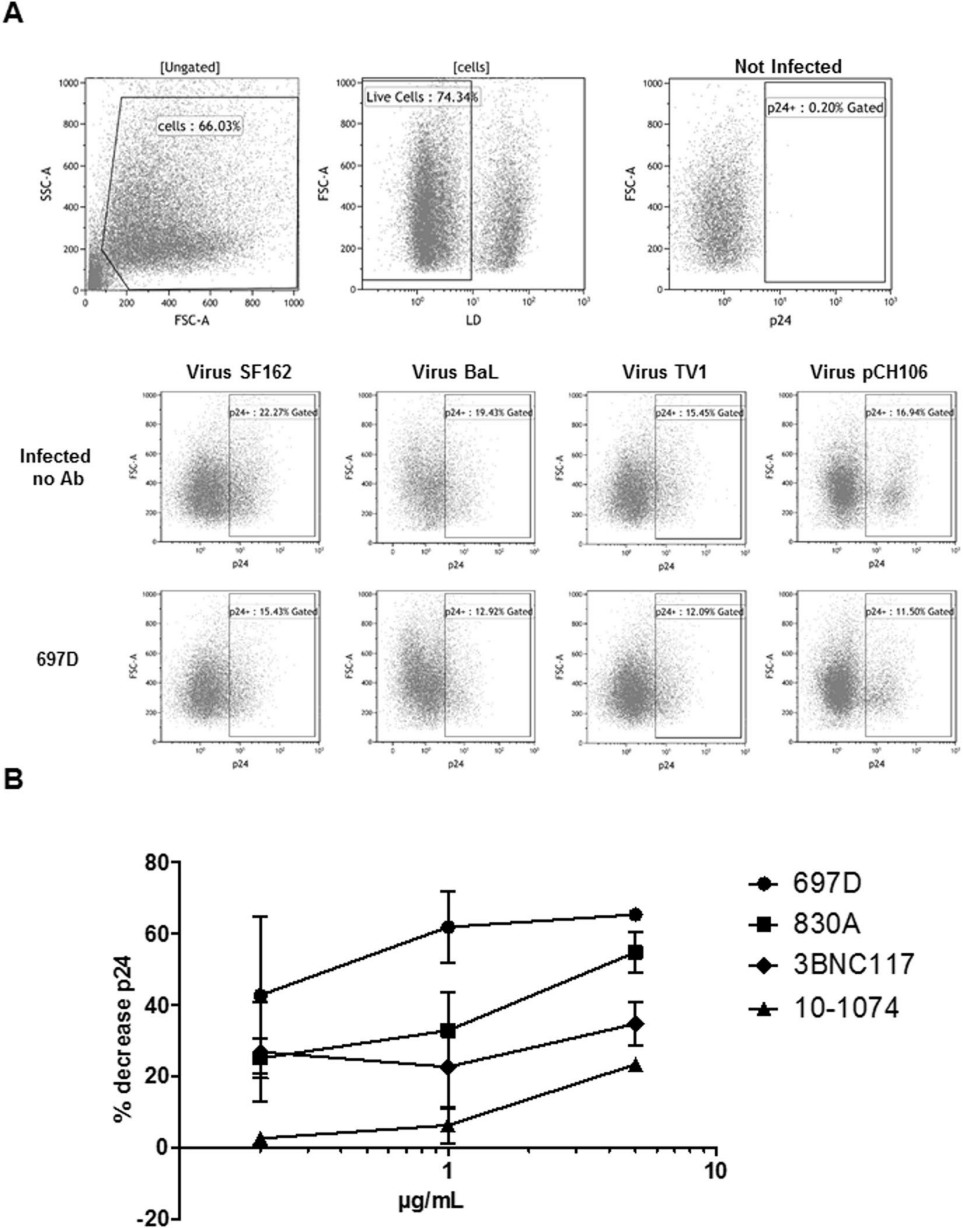
Flow cytometry data of three ADCC experiments. The infected cells were stained with a Live/Dead marker to exclude the dead cells from the analysis. After permeabilization, cells were stained for intracellular p24. The addition of 5 µg/ml of each mAb induced the lysis of infected cells, visible in the decrease in p24 positive cells. Data for 3 independent experiments with 3 different donor cells infected with different HIV subtypes are shown. (**A**) A dose-response curve is shown for mAbs to determine the best concentration for inducing NK cell mediated killing of HIV-1_SF162_-infected cells. MAbs were used at 5 -1- and 0.2 µg/ml and were specific for V2i (697D and 830A), CD4bs (3BNC117) and the V3-glycan (10–1074). The mean with SEM of one experiment in duplicates is shown (**B**).

The ADCC assay that we perform uses primary CD4 T cells infected with HIV-1 isolates produced by primary PBMCs (target cells) and autologous purified NK cells (effector cells). We require a minimum infection rate of 15% for the target cells and record the decrease of infected cells over 4 hrs, hence measuring the direct lysis of infected cells in the presence of Abs (Fig. 1A). All measurements were performed with at least 2 different donor cells to ensure reproducibility. This assay gives a highly physiologically relevant reading of the decrease of infected primary cells by Abs.

An initial titration of the Abs was carried out. We measured a dose response for all mAbs and selected the concentration that induced the highest NK cell mediated killing of HIV-1_SF162_-infected cells (Fig. 1B). Using this concentration (5 µg/ml) we then compared the ADCC activity of several bNAbs with mAbs with no or low neutralizing activity (here referred to as non-neutralizers). The mAbs used target several gp120 epitopes as indicated in Table 1.

**Table 1:** Human mAbs used.

mAb	Specificity	Source	Neutralizing Activity	Reference	Derivation
697-D	V2i	SZP	−	15	hybridoma
830A	V2i	SZP	−	50	hybridoma
1361	V2i	SZP	−	51	hybridoma
1393A	V2i	SZP	−	50	hybridoma
2158	V2i	SZP	−	49	hybridoma
2297	V2i	SZP	−	14	hybridoma
CH58	V2p	XPK	−	38	recombinant
CH59	V2p	XPK	−	38	recombinant
PG9	V2q	HIV Rep	**++**	33	recombinant
7B2	gp41 (cluster I PID)	HIV Rep	−	52	hybridoma
3BNC117	CD4bs	HIV Rep	+++	53	recombinant
10–1074	V3-glycan	HM	+++	54	recombinant
10E8	gp41 (MPER)	HIV Rep	**++**	48	recombinant
3BC176	gp41 (interprotomer)	HM	**+/**−	55	recombinant

SZP: Susan Zola-Pazner, XPK: Xiangpeng Kong, HIV Rep: NI H HIV Repository, HM: Hugo Mouquet.

**Figure 2:**
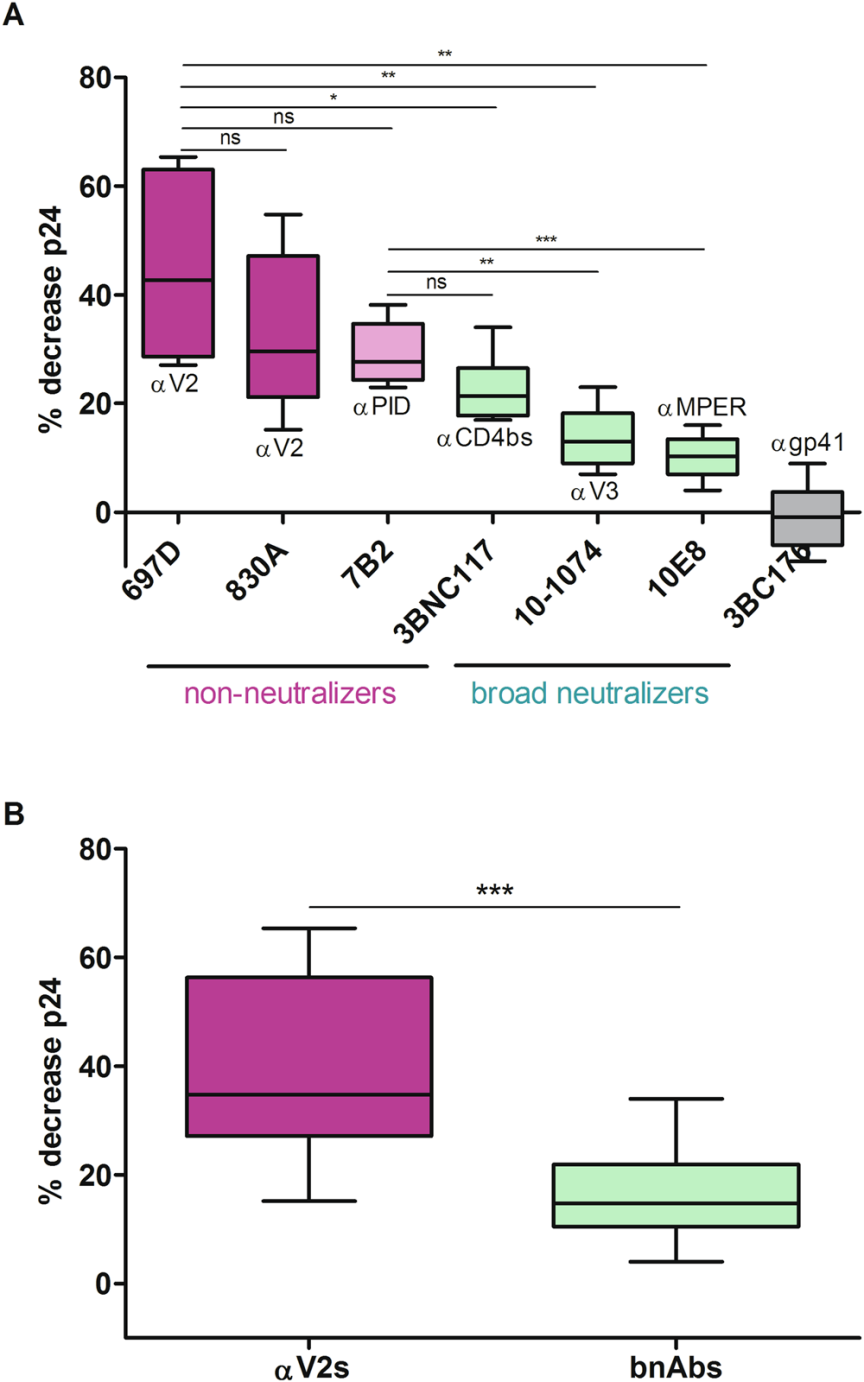
Antibody-dependent cell cytotoxcity (ADCC) with enriched primary CD4 T cells infected with HIV-1_SF162_. (**A**) ADCC activity of a set of mAbs was analyzed using 5 µg/ml of each mAb, using autologous NK cells as effector cells (E/T ratio = 1:1). A minimum of 5 different donor cells was tested per mAb. The percentage of target cell lysis (% decrease p24) was normalized to control wells without mAbs using the following formula: ((%p24 infected target cells with effectors − %p24 infected target cells with effectors and mAb)/ %p24 infected targets with effector cells) × 100. (**B**) Pooled ADCC results of mAbs 697D and 830A (V2s) and 3BC117, 10-1074, and 10E8 (bnAbs). A two-tailed unpaired ttest was used for statistical analyses.

We found that the non-neutralizing mAbs directed against the V2i epitope and the gp41 principal immunodominant domain (PID) were more potent in inducing NK cell mediated killing of HIV-1_SF162_-infected cells compared to the broadly neutralizing mAbs tested (Fig. 2A). Notably, the V2i mAbs reached high levels of ADCC, achieving levels of p24+ cell decrease of up to 55–65% for non-neutralizing mAbs 697D and 830A. As level of ADCC activity varied according to the primary donor cell used, we repeated the ADCC experiment 6 times with different PBMC donors. The mean of decrease of p24+ cells was 45% for mAb 697D and 33% for mAb 830A. The gp41 PID mAb 7B2, which is also non-neutralizing, achieved a maximum ADCC level of 38% and a mean of 29% when tested in 5 independent experiments with different PBMC donors. There was no statistically significant difference in the ADCC levels of these 3 non-neutralizers (Fig. 2A).

In contrast, the ADCC activity of the bNAbs reached maximum levels of 34%, 23%, and 16% for 3BNC117, 10-1074, and 10E8, respectively (Fig. 2A). They were each tested in 5–6 different ADCC experiments and the mean reduction in p24+ cells was 23%, 14%, and 10% for 3BNC117, 10-1074, and 10E8, respectively. This was significantly lower than the ADCC activity displayed by 697D. Finally, mAb 3BC176, targeting an interprotomer epitope of gp41, displayed no ADCC.

When comparing the reduction in p24+ cells induced by the 2 mAbs targeting the V2i epitope to the reduction achieved by the 3 bNAbs we found that the V2i mAbs were significantly better at inducing ADCC (p < 0,0001) on SF162-infected cells. The mean p24+ cell decrease was more than 2-fold higher with the V2i mAbs (39% for V2i mAbs vs, 16% for bNAbs) (Fig. 2B).

### V2i mAbs induce cross-clade NK cell mediated lysis

The high ADCC activity of the V2i mAbs demonstrated in our experimental conditions encouraged us to take a closer look at a panel of V2i mAbs (Table 1). We have previously shown that these V2i mAbs have little neutralizing capacity^12^. To further assess mAbs that target other epitopes in V2, we included in the study the V2p mAbs CH58 and CH59, isolated from RV144 vaccinees receiving subtype A/E and B gp120s, which had been shown to have weak and narrow neutralizing activity^32^ and therefore are categorized here as non-neutralizers (Table 1). Monoclonal Ab PG9, a V2q mAb that was isolated from a clade A donor, recognizes a quaternary epitope that requires the presence of the N160 glycan on gp120 and is a potent and broad neutralizer^33^ (Table 1).

**Figure 3:**
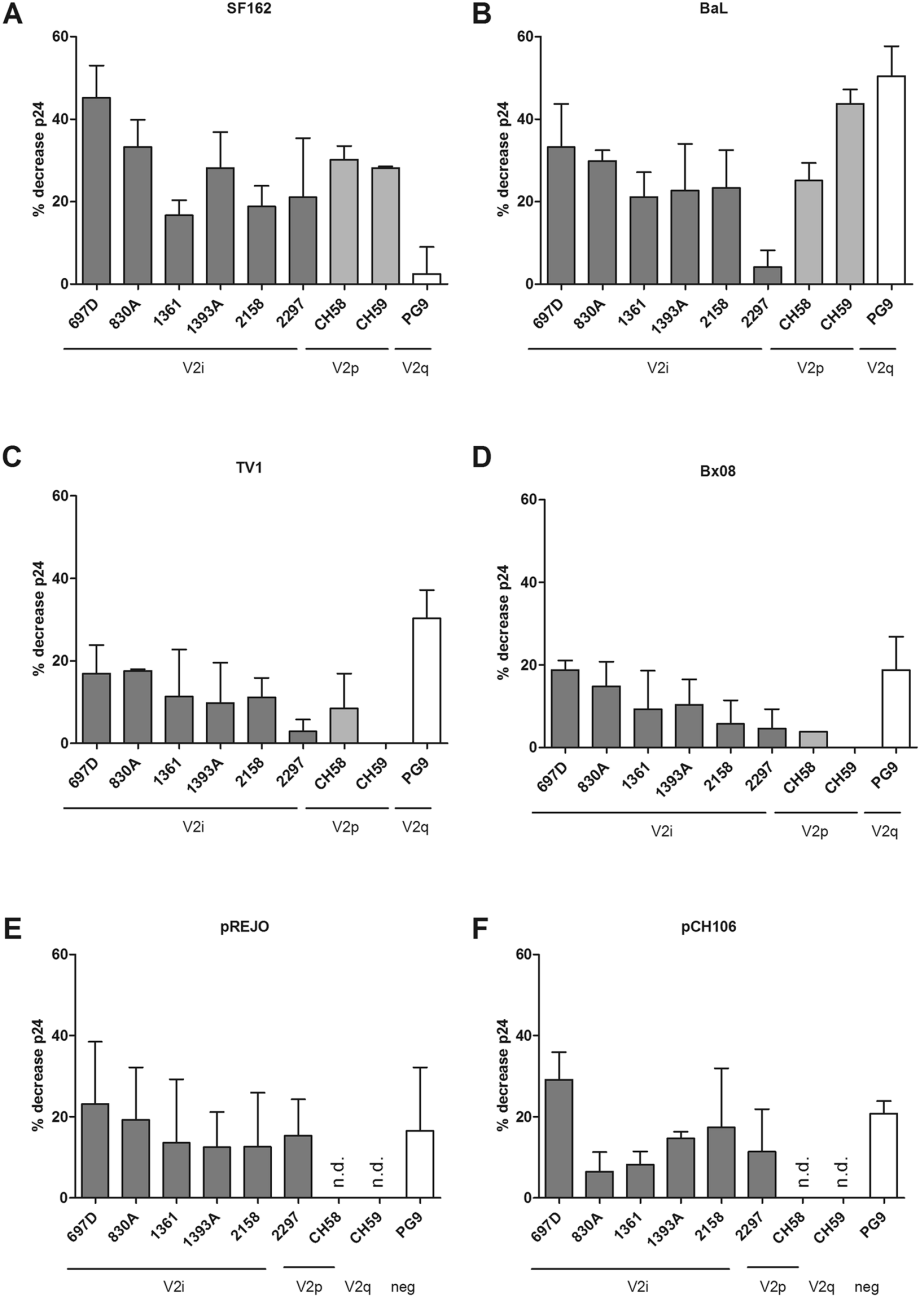
Antibody-dependent cell cytotoxcity (ADCC) on enriched primary CD4 T cells infected with SF162 (**A**), BaL (**B**), TV1 (**C**), Bx08 (**D**), pREJO (**E**), or pCH106 (**F**). ADCC activity of mAbs targeting the V2 region of gp120 was analyzed using 5 µg/ml of each mAb, using autologous NK cells as effector cells (E/T ration = 1:1). The mean of 2–3 independent experiments is shown with SEM. The percentage of target cell lysis was normalized to control wells without mAbs using the following formula: ((%p24 infected target cells with effectors − %p24 infected target cells with effectors and mAb)/ %p24 infected targets with effector cells) × 100.

All six tested V2i mAbs showed strong ADCC activity against SF162, as did the two V2p mAbs CH58 and CH59. Monoclonal Ab 697D induced the strongest decrease in intracellular p24+ cells (45%), followed by 830A (33%) and 1391 A (28%). CH58 and CH59 induced a 30% and 28% decrease in p24+ cells, respectively. PG9 did not induce ADCC against SF162 infected cells since the SF162 Env lacks the required N160 glycan^34,35^ (Fig. 3A). Furthermore, V2i mAbs were able to lyse CD4 T target cells infected with the clade B BaL virus, although with slightly lower ADCC activity (range of 4% to 33% of intracellular p24+ cell decrease). Both V2p mAbs CH58 and CH59 also strongly induced NK cell mediated killing of BaL infected cells with a mean 25% and 44% reduction in p24+ cells, respectively. PG9 displayed the strongest ADCC capacity against BaL with more than 50% of p24+ cell reduction (Fig. 3B). Low levels of ADCC were recorded when the cells were infected with TV1, a clade C virus and Bx08, a clade B virus, both classified as Tier 2 for neutralization. Against TV1, the maximum ADCC activity by V2i mAbs was detected with mAbs 830A and 697D, achieving a decrease in p24+ cell of 18% and 17%, respectively. V2p mAb CH58 induced ADCC levels of 8% whilst CH59 did not display ADCC activity against TV1 infected target cells. The V2q mAb PG9 performed very well in inducing ADCC against this subtype C, Tier 2 primary isolate with a mean of 30% reduction of p24+ cells (Fig. 3C). Similar levels of ADCC were achieved against Bx08, with maximum ADCC levels reaching and 19% (PG9 and 697D) (Fig. 3D). In addition, we also tested two Transmitted/Founder (T/F) viruses: all of the V2i mAbs exhibited ADCC activity against pREJO, with means ranging from 23% (697D) to 13% (1393 A and 2158). Similar ADCC levels were reached by the neutralizing Ab PG9 that induced a mean 17% decrease in p24+ cells (Fig. 3E). 697D showed an ADCC activity of 29% using T/F virus pCH106, compared to 21% for PG9 (Fig. 3F).

Overall, the V2 mAbs consistently induced NK cell mediated killing of primary cells infected with a variety of different viruses.

### Binding to infected cells is necessary but not sufficient for ADCC activity

**Figure 4:**
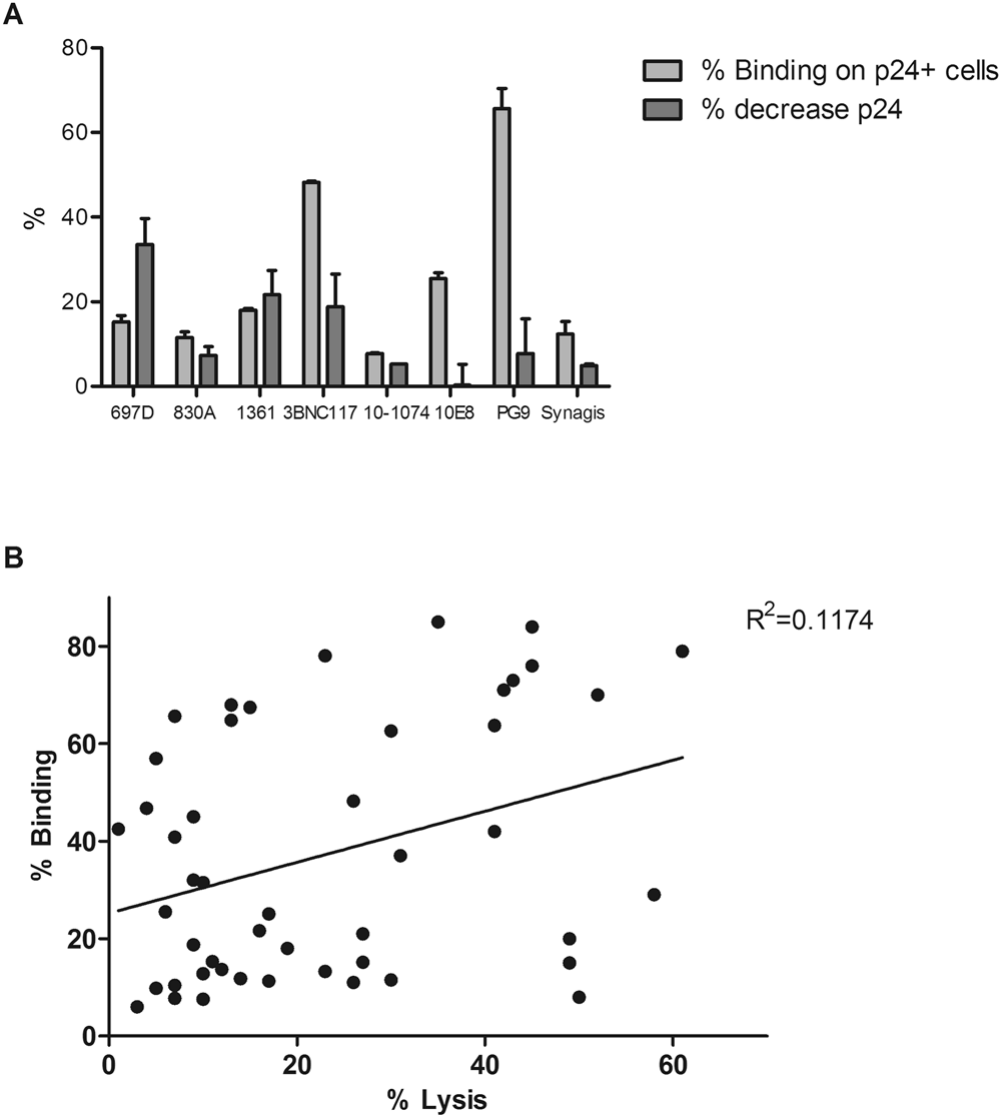
Binding of mAbs to enriched primary CD4 T cells infected with pREJO. Binding of mAbs targeting different regions of gp120 was analyzed by incubating 5 µg/ml of each mAb for 4 hrs with infected primary cells. At the same time, ADCC was measured in parallel. Mean of one representative experiment in duplicates with SEM is shown (**A**). The correlation of binding versus ADCC activity for the same set of mAbs (697D, 830A, 1361, 3BNC117, 10-1074, 10E8, PG9) was calculated by combining the results of 3 independent experiments, using the Pearson two-tailed correlation test. p = 0.0171 (**B**).

Abs need to bind to infected cells in order to carry out ADCC. We therefore measured the capacity of Abs to bind p24+infected cells. V2i mAbs were able to bind p24+ pREJO-infected cells at levels ranging between 12% and 18%. This suggests that V2 epitopes are expressed on primary infected cells. However, this binding was less efficient than that observed with some bNAbs, which reached levels of up to 66% (Fig. 4A). Moreover, the binding capacity of mAbs to infected cells did not strongly correlate with their ADCC activity (p = 0.0171) (Fig. 4B). Indeed, binding is required but not sufficient to carry out the killing of infected cells and the triggering of additional steps via the Fc domain is necessary to induce ADCC.

## Discussion

This is the first report showing that non-neutralizing anti-V2 mAbs elicited in HIV-1 infected patients have strong ADCC activity. Cross-reactive ADCC capacity was observed for four subtype B and C primary isolates (SF162, BaL, Bx08, and TV1) and two subtype B Transmitted Founder infectious molecular clones (pREJO, pCH106).

Abs displaying ADCC activity have to recognize and bind infected cells via their Fab fragments. Indeed, we found that anti-V2 mAbs bind to the V2 epitopes displayed on the surface of infected cells. However, the precise accessibility of V2 epitopes required for binding to infected cells was not determined. It has been suggested that “transitional epitopes” emerge on the surface of infected cells during conformational rearrangement^12,36^. Moreover, epitopes may vary according to their stage of infection (early infection versus latently infected cells, cells covered with virus particles versus cells in the course of virus budding). Binding of CD4 was also shown to downregulate the expression of certain epitopes. Thus, the nature of the epitopes of the viral envelope glycoproteins exposed on the surface of these infected cells requires further investigation. We suggest that the V2 loop epitopes remain accessible on infected primary cells and that Abs targeting these epitopes are therefore highly relevant for ADCC activity under physiological conditions.

Previously, mAbs CH58 and CH59 were shown to induce ADCC using an NK cell line as effectors and a T cell line (CEM) infected with the IMC AE.CM235, the same subtype as the immunogen used for immunization^32,37,38^. Here we show that CH58 and CH59 mAbs also display ADCC activity against SF162 and BaL infected primary cells.

The reported ADCC activity of mAb PG9 is contradictory depending on the assay used. With primary cells and primary BaL and TV1 virus, we detected high ADCC activity of PG9. Previously, no ADCC was observed using this mAb with the HIV-1_JRCSF_-infected CEM.NKR.CCR5 cell line and primary NK effector cells whilst others reported ADCC when CEM.NKR.CCR5 cells infected with HIV-1_NL4-3_ and HIV-1_JR-FL_ and an NK cell line expressing human FcγRIIIA was used as effector cells. The binding of the Fc domain of PG9 and other Abs to the FcR on the surface of different effector cells may explain the distinct results^39,40^.

Abs have to capture effector cells via their Fc domain to trigger the ADCC process. Huang *et al*. recently showed that the mAb b12, mutated in the Fc domain to modify its Fc binding capacity, displayed distinct ADCC, or phagocytic functions^41^. These results firmly demonstrate that binding to the Fc domain is decisive for Fc-mediated functions. In this regard, binding to the tip of the envelope trimeric spike, where V1/V2 epitopes are located, may facilitate binding of the effector cell via the Fc domain of the Ab.

Noteworthy, in our study, the binding of the V1/V2 Abs did not strongly correlate with cell lysis implying that the Fc domain participates in induction of lysis. All V2i mAbs used here are IgG1, except for 830A, which is IgG3. The mAbs tested herein were both recombinant and hybridoma-derived; the Fc domain is that of the Ab from the infected patients for the V2i mAbs but recombinantly engineered to be IgG1 for the bNAbs^14,42^. Therefore, the specificity and derivation of the Abs studied here differed from those examined by Bruel *et al*.^31^, who used different mAbs constructed with identical heavy chains. With their panel of mAbs, they found that non-neutralizing Abs targeting V3 and the CD4 binding site of gp120 and the PID of gp41 displayed little to no detectable ADCC activity on reactivated cells isolated from HAART-treated HIV-infected patients^31^. Of note, ADCC activity with mAbs of these specificities but with distinct Fc domains has previously been described^43,44^. Whether this discrepancy is due to the Fc domain of the Abs needs to be further assessed.

Since the Fc domains of the patient-recovered V2i mAbs were able to induce NK cell-mediated killing, they are able to efficiently trigger the FcγRIIIA (CD16), which is expressed on primary NK cells. We therefore propose that the combination of the heavy and light chains that constitute these V2i mAbs are particularly favorable for inducing ADCC, and that increased attention be given to the characterization of the Fc domains of Abs, since they are essential for the induction of ADCC^45^.

Overall, our results revealed ADCC activity of non-neutralizing anti-V2 Abs isolated from HIV-1 infected patients. Notably, these V2i mAbs show specificities that are similar to the Abs that correlated with reduced infection rates in the RV144 vaccine trial, and the V2i mAbs displaying ADCC activity are further similar to the Abs induced in RV144, where reduced infection was correlated with ADCC rather than neutralizing activity. Further significance to the studies described above comes from the physiologically relevant ADCC assay, which employed autologous primary effector cells and primary target cells infected with primary isolates and transmitter/founder viruses. These results emphasize the potential role of anti-V1/V2 Abs for ADCC activity and further suggest that lysis of infected cells may be an additional relevant antibody inhibitory effect that we should continue to unravel to define the components required for a protective vaccine.

## Materials and Methods

### Cells and viruses

Peripheral blood mononuclear cells (PBMCs) were isolated from whole blood via Ficoll-Hypaque sedimentation. The blood was received from healthy volunteers at the Blood Transfusion Center (EFS) in Strasbourg. Using an AutoMACS (Miltenyi Biotec) for immunomagnetic bead isolation, NK cells were separated from the PBMCs via anti-CD56 beads and then frozen in liquid nitrogen. The rest of the cells were depleted of CD8^+^ cells (anti-CD8 magnetic beads) to obtain enriched primary CD4^+^ T cells, which were then activated for 3 days using phytohemagglutinin (PHA). The activated cells were infected with purified and concentrated primary HIV-1 isolates (SF162, BaL, or TV1) for 72-96 hrs. Infection efficiency was analyzed after 2 days via flow cytometry and the cells were used if the levels of intracellular p24 were above 15%.

### ADCC

The ADCC assay was performed using primary cells and primary HIV-1 isolates, as previously described^24^. Briefly, NK cell-depleted PBMCs were infected for 4 days with primary isolates. Our ADCC assay is highly challenging as experiments were carried out only if at least 15% of primary CD4 T cells were infected (p24+) after 4 days. 2 × 10^5^ HIV-1 infected enriched primary CD4+ T cells were plated in 50 µl of RPMI-1640, 10% FCS, and 50IU interleukin-2 (R&D Systems) in a U-bottom 96-well-plate and mixed with 2 × 10^5^ autologous NK cells in 50 µl of the same medium (1:1 effector: target ratio). 5 µl CD107a-PE (Milteny Biotec) was added per 1 × 10^6^ autologous NK cells. 25 µl of mAbs at the indicated concentrations were added. After 4 hrs of incubation, cells were stained using Live/Dead Fixable Violet Dead Cell Stain Kit (Life Technologies), CD3-APC Vio770 (Miltenyi Biotec), CD16-PercP Vio700 (Miltenyi Biotec), and CD56-APC (Miltenyi Biotec). Cells were then fixed and permeabilized (Cytofix/Cytoperm; BD Biosciences) and stained for intracellular p24 (KC57-RD1-FITC; Beckman Coulter) to measure infection rate. The percentage of target cell lysis was normalized to control wells without mAbs using the following formula:

((%p24 infected target cells with effectors − %p24 infected target cells with effectors and mAb)/ %p24 infected targets with effector cells) × 100. This takes into account the decreased infection rate due to direct lysis in the absence of Abs.

Less than 1% of variation within the 4 control wells was observed, demonstrating that the infection rate is highly homogeneous. Due to the complexity of this assay, a variability of the lysis capacity was observed depending on the different donor cells used. We repeated each experiment with 2 to 6 different donor cells according to the assay performed. Although the lysis intensity varied, the general pattern of ADCC activity for the different Abs was constant, demonstrating reproducibility.

### mAbs

The mAbs used are listed in Table 1.

### Statistics

For all experiments, the mean of at least one experiment in duplicates with SEM is shown (Figs 1 and 4: one experiment in duplicates, Fig. 2: 5–6 experiments in duplicates, Fig. 3: 2–3 experiments in duplicates). For statistical analyses in Fig. 2, a two-tailed unpaired ttest was carried out. The correlation of Fig. 3 was calculated by combining the results of 3 independent experiments and using the Pearson two-tailed correlation test.

### Ethics Statement

Human peripheral blood mononuclear cells were purified from blood packs purchased from the blood bank (Établissement Français du Sang, Strasbourg), with written informed consent from the donors.
